# Making Common Sense of Vaccines: An Example of Discussing the Recombinant Attenuated *Salmonella* Vaccine with the Public

**DOI:** 10.1007/s11569-014-0198-6

**Published:** 2014-07-10

**Authors:** Dorothy J. Dankel, Kenneth L. Roland, Michael Fisher, Karen Brenneman, Ana Delgado, Javier Santander, Chang-Ho Baek, Josephine Clark-Curtiss, Roger Strand, Roy Curtiss

**Affiliations:** 1 The Centre for the Study of the Science and the Humanities, University of Bergen, Allégaten 34, Post Box 7805, 5020 Bergen, Norway; 2 The Biodesign Institute, Center for Infectious Diseases and Vaccinology, Arizona State University, 1001 S McAllister Avenue, Tempe, AZ 85287 USA; 3 Nucleus for Microbiology and Immunity, Center for Genomics and Bioinformatics and The School of Life Sciences, Universidad Mayor, Chile and Arizona State University, Camino la Piramide 5750, Huechuraba, 8580745 Chile; 4 Synthetic Biology R&D, Bioscience Division, Life Science Solutions Group (LSG), Thermo Fisher Scientific, 5791 Van Allen Way, Carlsbad, CA 92008 USA

**Keywords:** Biotechnology, Ethics, Legal issues, Social aspects, Society, Societal values, Trust, Vaccine, Values

## Abstract

Researchers have iterated that the future of synthetic biology and biotechnology lies in novel consumer applications of crossing biology with engineering. However, if the new biology’s future is to be sustainable, early and serious efforts must be made towards social sustainability. Therefore, the crux of new applications of synthetic biology and biotechnology is public understanding and acceptance. The RASVaccine is a novel recombinant design not found in nature that re-engineers a common bacteria (*Salmonella*) to produce a strong immune response in humans. Synthesis of the RASVaccine has the potential to improve public health as an inexpensive, non-injectable product. But how can scientists move forward to create a dialogue of creating a ‘common sense’ of this new technology in order to promote social sustainability? This paper delves into public issues raised around these novel technologies and uses the RASVaccine as an example of meeting the public with a common sense of its possibilities and limitations.

## Introduction

The last decades, many advances in biology have been boosted by crossing biology with engineering. Recombinant DNA techniques have made this possible, and are a technical foundation for the creation of new products, which may be aimed to benefit society. An example of this lies in the design, development and creation of vaccines.

Vaccines, especially in the United States, can be a very contentious object in societal circles, although their benefits have proven to be very effective for overall public health. This is partially due to societal values. It is therefore important that research products, whether they be vaccines or among the plethora of other methods or products, whose goal is to improve society in one way or another, take seriously the idea of ‘social sustainability’. But how?

This paper is an academic contribution of how the life sciences and humanities can describe and frame, and thus understand, their products of research with and for society. The first part of this paper reviews public trust and distrust in society. We then use the case of the Recombinant Attenuated *Salmonella* Vaccine to illustrate how scientists can respond to societal questions of value as first steps to dialoguing with society for biotechnologies that are socially and scientifically robust.

### Making Common Sense of Vaccines: Public Trust and Distrust in Science

There are several sources of public trust in science. First, the public may see science and the scientific method as a provider and guarantor of truth or at least valid, reliable and objective knowledge. Second, they may experience that science helps them meet their needs and improves health and welfare. Third, the scientific ethos invites trust: the honest, open, unselfish and self-critical pursuit of knowledge for the common good [1, 2].

Sources of distrust are equally many. The history of vaccines is riddled with public controversies from its inception until today. For instance, through miscommunication or blatant falsification of data the notion that the measles, mumps, rubella (MMR) vaccine causes autism in children has become pervasive, resulting in fewer parents vaccinating their children [3–5]. Similarly, controversy over the anthrax vaccine has prompted many armed servicemen and women to decline vaccination [6–8]. From the perspective of scientists, vaccine opponents may occasionally appear as misinformed and ignorant^1^^,^ 2 [9]. If we take the perspective of the opponents, however, the distrust can be quite understandable. They or their child may have experienced something they believe to be an adverse effect of the vaccine, as in the well-publicized Jenny McCarthy case,^3^^,^ 4 ^,^ 5. Vaccine dissenters may have encountered disagreement or conflicting messages from different physicians, or arrogant representatives of the medical and scientific community when raising their issues. They may also be worried about the economic or political dimensions of vaccine programs, in particular if the distrust in science is linked to a similar distrust in government. In such a situation, there might be a need to improve public understanding of the technicalities of science. We believe, however, that there is an equal need to improve scientists’ understanding of how the different publics perceive the broader issues in which science is an element. Accordingly, in this paper we introduce the notion of ‘*common sense*’. The Cambridge Dictionary definition of common sense is: ‘the basic level of practical knowledge and judgment that we all need to help us live in a reasonable and safe way’^6^ This can be understood as shared understanding, and understanding that has been made on common grounds and is acceptable for all. ‘Common grounds’ means a meeting between parties that may differ in their interests and values but who are willing to negotiate them. This is precisely the condition for common acceptance and trust.

### How to Make Common Sense of Vaccines?

For scientists, this means that there are several tasks in the science-society interface. First, there is the responsibility to establish an effective science communication about what is known, what is unknown and what remains uncertain and to what extent. Secondly, the scientific community should prove its commitment to the scientific ethos by its quality of communication with the public: through accountability, transparency and openness, including their own motives and interests. The Asilomar conference in 1975 serves as a source of inspiration in that respect; an example of how scientific initiatives can inform the public and prevent misconceptions from stifling scientific progress [10]. In addition, scientists must be aware that we are now living in an era in which trust must be earned and in which the public does not accept claims to knowledge simply because they are made by scientists. This development is a fact, for better or for worse.

### Research on Public-Science Dialogues

Bean [11] provides a useful overview of concerns voiced at anti-vaccine web sites. We use these questions to begin the informed dialogue of the RASVaccine, in addition to two socio-cultural communication techniques.

Decisions by members of the public on what science is ‘good’ and what science is ‘bad’ are not and cannot be solely based on facts; values, ideals of what is useful or important for an individual, are at least as significant. Values also refer to a person’s sense of behavioral, moral and ethical norms. Values can be a result of a person’s cultural identity. Kahan et al. [12: 148] put forward a definition of ‘cultural cognition’: ‘the tendency of individuals to fit their perceptions of risk and related factual beliefs to their shared moral evaluations of putatively dangerous activities.’ In other words, scientific debates may become discredited if a person or social group perceives the debate as grounded in an ideal or value culture with which they do not agree. For example, if the people with whom I identify and who are important to me think the world is flat, I, too, will adopt that belief, despite facts that prove otherwise, to maintain my social status and avoid social dissonance among my peers.

Kahan [13] outlines two approaches to societal debates that are polarized by cultural cognition and disconsensus. The first, based on the research of Cohen et al. [14], is to ‘present information in a manner that affirms rather than threatens people’s values’ and the second, ‘to make sure that sound information is vouched for by a diverse set of experts’ [13: 297].

### Why do We Need Common Sense of Vaccines?

Vaccines have become so routine in modern life that their significance for public health may easily be forgotten. Two centuries of scientific research and vigorous prophylactic inoculation programs laid the foundation for the eradication of the disfiguring and deadly disease smallpox (1979) [15] and the control of the spread of polio [16–18], mumps, measles, rubella and other contagious diseases. [19–22]. With the current rate of progress in molecular biology, it is likely that the repertoire of effective vaccines will continue to expand. It is important therefore, that novel vaccine technologies be introduced on ‘common grounds’.

The successful introduction of new promising vaccines depends upon a lot more than science. More than most technologies, vaccines require acceptance on a collective level, since the objective is disease control in the population. Collective acceptance and legitimacy, however, is a factor that cannot be taken for granted in vaccination programs or indeed any other emerging biotechnology. To gain insight into this matter, we review some of the sources of public trust and distrust and discuss scientists’ responsibilities with regard to trust. We suggest a road ahead by indicating how a more dialogical form of science communication might bridge gaps of understanding and create shared perspectives upon – a ‘common sense’ of – vaccines.

The public debate about the perceived risks of vaccines is contentious and scientists point to current communication on vaccines as potentially counterproductive [23, 24]. How can we as scientists begin to understand the core issues such as public trust and values as we participate in this debate? This paper is the product of a collaboration between scientists studying the ethical, legal and social impacts (commonly known as ELSI) of new technologies and those scientists involved in the design and creation of these new technologies; to be specific, a new type of vaccine that is embedded in the DNA of a bacteria. This paper has two parts in the goal of defining a ‘common sense’ of the public concerns and actual risks of vaccines. First, the ELSI scientists from the University of Bergen put forward arguments in a focus group of vaccine scientists on why scientists and the public need to make a ‘common sense’ understanding of new technologies that will have a societal impact. The ELSI scientists proposed 7 specific questions with which the public is likely to be concerned. Then the vaccine scientists from Arizona State University responded to these questions in an effort to make common sense of their specific research product, a Recombinant Attenuated *Salmonella* Vaccine (RASV) to prevent pneumonia. Thus, this paper represents one example of many possible avenues of public-science dialogue developed through interdisciplinary collaboration.

## Method

To address this issue, we focus on information about the RASVaccine using Bean, Cohen et al. and Kahan’s insights in an example of scientists responding to the public. Since RASV is a live, *Salmonella*-based vaccine, it should not come as a surprise if lay people might distrust the RASV. Rather than a public misunderstanding of science, this is established common sense. For the public, the obvious question that follows is: how can a bad bug possibly become a good bug? This is on one hand a question about the scientific competence and ethos, which has to be displayed as transparency and willingness to communicate the science in accessible language. On the other hand, common sense should be established on a societal level: motives, purposes and values informing the research, as well as needs, concerns and other issues voiced by publics and citizens. This will be the point of departure for dialogue.

The ELSI researchers reviewed the literature for primary references that describe the public in relation to perceptions of science through values. From this review, we represent the ‘public’ through five questions that people who visit anti-vaccine websites have had [11]: 1) What is the need for the RASV? 2) What are some alternative treatments? 3) What are the safety issues? 4) Who profits from the RASV? 5) How would the RASV affect my civil liberties? In addition, one question referring to Cohen et al. [14] that affirms the value of trust 6) Why should I trust the scientists working on these things? and finally a question referring to Kahan [13] that addresses a broad-backing from experts and relate them specifically to the Recombinant Attenuated *Salmonella* Vaccine: 7) How can I know that the public health institutions are doing their best for me and my safety? During a 3-week in-lab visit by the ELSI researchers from the University of Bergen in October 2010, the ELSI researchers mediated a series of ‘dialogue sessions’ on various ELSI topics in which the RASVaccine scientists were interested. In collaborations after the in-house visit, these seven questions were put forward to RASVaccine researchers by the ELSI researchers. Consensus answers from scientists are provided and discussed in the sections below.

### What is the Need for the RASV?

Infectious diseases are rampant throughout the developing world. The goal of the RASV approach is a low cost, oral (needle-free) vaccine designed to induce long lasting immunity.

RASV is special because it stimulates all three arms of the immune system, humoral (antibodies), mucosal and cellular, while while injectable vaccines are very efficient at stimulating humoral immunity, but are typically poor at stimulating cellular and mucosal responses. One of the scientists in the author team expressed it as follows: ‘RASVs are very inexpensive to manufacture, with expected costs to be less than $0.50 a dose’ [25: 9]. (The authors note that deploying the vaccine into the field - e.g. transportation and cold chain requirements - would necessarily add additional costs.) Our goal is to make the vaccine available to local manufacturers in the developing world willing to make only a small profit.’

### What are Some Alternative Treatments?

There are many needle-based vaccines available in the US and Europe that can be used to prevent diseases prevalent in the developing world, such as BCG for tuberculosis and conjugate vaccines and pneumococcal polysaccharide vaccines for bacterial pneumonia. The cost of these vaccines is heavily subsidized to make them affordable in the developing world [26]. An alternative to disease prevention by vaccination is treatment with inexpensive antibiotics/antimicrobials. However, bacteria can rapidly develop resistance to such compounds [27]. Additionally, this would require a major shift in policy from drug manufacturers and governments to invest in the production of novel antimicrobials [28]. There is little economic incentive to do this and many pharmaceutical companies have ceased research to discover and develop new antimicrobial agents, with the exception of anti-viral agents [29].

### What are the Safety Issues?

*Salmonella* Typhi can cause diseases such as typhoid fever. Many of the genes necessary for virulence have been identified. The current RASV is an *S*. Typhi strain that has 10 different mutations designed to eliminate the ability of *Salmonella* to cause disease and related symptoms of discomfort [30] and to enhance its ability to induce a protective host immune response.

Other safety concerns have guided the construction and design of the RASV. These include bio-containment of the vaccine, such that it does not spread from the patient receiving the vaccine, transfer of exogenous genes from the vaccine strain to the natural gut flora, and potential inoculation of immune compromised individuals.

One of the side-effects that can occur with live attenuated vaccines is a back-firing, or reversion to virulence. Reversion is the standard boogeyman for live vaccines. To address this concern, we use complete gene/operon deletions to make the odds of reversion extremely small. Another danger is that a live vaccine will be able to interact with other microbes in the host and/or environment and either pass traits to them or acquire new abilities from them (thus altering vaccine behavior). While theoretically possible, based on what we know about the frequency of gene transfer, the possibility of this happening with our RASV strains, containing multiple genetic alterations, is statistically insignificant.

The safety of newborns, infants, pregnant mothers, the elderly, malnourished, people infected with HIV or *M. tuberculosis* or who are or otherwise immunocompromised is also an important issue. These groups can be particularly at risk and may respond differently to RASV than normal, healthy adults.

Given these risk and safety issues, which may be the sources of public trust in the RASV? We will point at three sources that we think provide a good point of departure for dialogue. First, as scientists we have to demonstrate our moral commitment to the safety of our vaccine. Perhaps our main contribution to the public debate is to express our commitment and show our efforts to design our strains and our pre-clinical and clinical studies to address these issues.

Secondly, we have to claim expertise and explain its basis in the institution of peer review. While there is no direct way one can know whether a particular scientist can be trusted, what we can put on the table are the reasons to believe in science as institution. Peer review has its problems and is not a truth machine; the philosophically informed reason to support it is basically that there is no better alternative.

Finally, there are the governmental institutional mechanisms of protective regulatory protocols to ensure the safety of the public and test the safety of the vaccine. For instance, one has to obtain preclinical and phase I, phase II, and phase III data on the specific population in order to satisfy the FDA’s rigorous standards as described. To make common sense around the safety issues would then mean to discuss in good faith our claims of expertise and moral commitment, and the reasons for believing in FDA and similar institutions.

### Who Profits from the RASV?

Our goal is prevention and eradication of infectious diseases for human and animals. Local governments and communities can also profit monetarily and socially. By decreasing the disease burden in developing countries, we can create a healthier, more efficient and productive workforce. This would lead to a healthier environment by reducing the environmental loads of targeted pathogens.

The eventual vaccine manufacturer will also profit from the RASV, since if they don’t make some sort of profit, it is unlikely they would want to manufacture the vaccine. Since RASVs are relatively inexpensive to manufacture compared to injectable vaccines, our goal is to facilitate the availability of the vaccine to local manufacturers in the developing world willing to make only a small profit. The Gates Foundation has covered all of the RASV research costs^7^ to date, including preclinical studies and a Phase I trial, thereby reducing the initial research and development costs to the eventual manufacturer. This also encourages investment in the technology while keeping the profit margin to a minimum with the explicit distribution condition that the vaccine be available to all at very low or no cost.

### How Would the RASV Affect Civil Liberties?

As mentioned above under Safety Issues, one problem with any live vaccine is that it can be spread through the population [31]. This can work out well, since you can get herd immunity (protection of unvaccinated individuals from contagious diseases due to the vaccination of a significant amount of surrounding individuals) without having everyone come in to the clinic, but it also means that those who shouldn’t be vaccinated (people who are at-risk for adverse reaction) and those who don’t want to be vaccinated could accidentally be exposed. Ethical standards now require informed consent. One scientist we’ve talked to on this issue stated: ‘I would consider it a civil liberties violation if I was given a drug I did not wish to receive without my consent, so I don’t think RASVs should be considered differently.’

Given the premise of a safe and efficacious vaccine, civil liberties would be less of an issue. Rather, vaccination would then be seen as a civic duty to protect oneself and one’s fellow citizens, on par with washing one’s hand when working in a kitchen or a hospital. In other words, common sense around civil liberties is intrinsically linked to the issue of safety.

### Why Should I Trust the Scientists Working on These Things?

Let us first say why we trust our fellow scientists. In the research and academic world, there are a number of checks and balances that are designed to build trust. All scientists developing vaccines must satisfy the FDA’s requirements before their vaccine can be licensed for use. Academic scientists are obliged to have their work evaluated by peer review before publication in academic or medical journals. We can never know for sure whether a particular scientist is trustworthy, but in general we do trust published peer-reviewed sources more than sources that do not use such checks (e.g. webpages, blogs, non-academic books).

We cannot, and nobody can, demand that others should trust us. Trust has to be earned and built through interaction. The reason to enter into dialogue is not to provide reasons why people should stop thinking for themselves and trust others. Rather it is to provide reasons that promote a mutual understanding that develops into trust - promote a common sense between previous opponents.

### How Can One Know That the Public Health Institutions Care for Citizens’ Safety?

Because public health policy is carried out by government agencies, they may be linked to a public distrust of government. Violations such as Tuskegee syphilis experiment [32] must be reminders that such distrust is not unfounded. While current standards for the use of human subjects in clinical trials include many safeguards covered in the Code of Federal Regulations Title 21 - informed consent, Institutional Review Boards and independent oversight committees^8^ - communication between the scientific and lay communities must be consistent and transparent. This requires a scientifically literate electorate and a dedication by scientists and policy makers to engage in outreach programs.

As in any situation, trust is earned through past deeds and accomplishments. To bolster public trust and to ensure the safety of volunteers, there are stringent requirements regulations during a vaccine clinical trial. Firstly, there is informed consent. Anyone in the study must be informed to the benefits and risks of the experimental procedures. Secondly, research is overseen by an independent internal regulatory board (IRB) made up of scientists, medical experts and members of the general public. These boards approve the design of the study and protect volunteers against violations of federal regulations. Lastly, an independent data and safety monitoring board (DSMB) reviews the safety information collected during the course of a clinical trial, and decides whether to continue a trial or halt it to protect the volunteers.

## Conclusions

This text operates on three important levels. First, we reflect on the need to improve the science-society dialogue in the realm of vaccines. Then, we give concrete examples of this dialogue by outlining legitimate questions from the public and answering them in our capacity as scientists in an effort to create a ‘common sense’ of the specific type of vaccine that we work with. Finally, we offer this approach to our science peers as one example of how to meet the public on highly contested topics like vaccines. Public trust in new technologies is needed in order to attain socially robust science and we encourage scientists to do their part and openly engage with the public in this two-way street.

We feel this paper is an important contribution to the literature as a guide to present information from the realm of biotechnology towards the public realm as a first step in making a ‘common sense’ of a technical object. As such, we hope the result of our ELSI-RASVaccine collaboration closes the communication gap Kahan [13] refers to by acknowledging and addressing cultural cognition and values of the public as a springboard to structuring an otherwise polarized and unpredictable debate about biotechnical products.

The philosopher Karl Popper expressed an essential scientific virtue in his saying: ‘I may be wrong and you may be right, and by an effort we may get nearer to the truth.’ Although misinformation may have dominated several of the vaccine controversies [33, 34], it remains a scientific responsibility to be open to the possibility that the critics and dissidents can be right the next time. We should also appreciate that it may require time and effort to understand their real concern.

## Footnotes

1
http://time.com/46914/vaccine-opponents-wrong/, Retrieved May 29, 2014.

2
http://www.nytimes.com/2014/05/09/upshot/vaccine-opponents-can-be-immune-to-education.html?_r=2,, Retrieved May 29, 2014.

3
http://content.time.com/time/magazine/article/0,9171,1968100,00.html,, Retrieved May 29, 2014.

4
http://www.huffingtonpost.com/jenny-mccarthy/whos-afraid-of-the-truth_b_490918.html,, Retrieved May 29, 2014.

5
http://www.wired.com/2009/10/ff_waronscience/all/, Retrieved May 29. 2014.

6
http://dictionary.cambridge.org/dictionary/british/common-sense. Retrieved May 22, 2014.

7 (Bill & Melinda Gates Foundation Grand Challenges in Global Health grant 37863)

8 see: http://www.fda.gov/scienceresearch/specialtopics/runningclinicaltrials/ucm155713.htm
